# Spatial Heterogeneity in Ecologically Important Climate Variables at Coarse and Fine Scales in a High-Snow Mountain Landscape

**DOI:** 10.1371/journal.pone.0065008

**Published:** 2013-06-07

**Authors:** Kevin R. Ford, Ailene K. Ettinger, Jessica D. Lundquist, Mark S. Raleigh, Janneke Hille Ris Lambers

**Affiliations:** 1 Department of Biology, University of Washington, Seattle, Washington, United States of America; 2 Department of Civil and Environmental Engineering, University of Washington, Seattle, Washington, United States of America; Institute of Botany, Czech Academy of Sciences, Czech Republic

## Abstract

Climate plays an important role in determining the geographic ranges of species. With rapid climate change expected in the coming decades, ecologists have predicted that species ranges will shift large distances in elevation and latitude. However, most range shift assessments are based on coarse-scale climate models that ignore fine-scale heterogeneity and could fail to capture important range shift dynamics. Moreover, if climate varies dramatically over short distances, some populations of certain species may only need to migrate tens of meters between microhabitats to track their climate as opposed to hundreds of meters upward or hundreds of kilometers poleward. To address these issues, we measured climate variables that are likely important determinants of plant species distributions and abundances (snow disappearance date and soil temperature) at coarse and fine scales at Mount Rainier National Park in Washington State, USA. Coarse-scale differences across the landscape such as large changes in elevation had expected effects on climatic variables, with later snow disappearance dates and lower temperatures at higher elevations. However, locations separated by small distances (∼20 m), but differing by vegetation structure or topographic position, often experienced differences in snow disappearance date and soil temperature as great as locations separated by large distances (>1 km). Tree canopy gaps and topographic depressions experienced later snow disappearance dates than corresponding locations under intact canopy and on ridges. Additionally, locations under vegetation and on topographic ridges experienced lower maximum and higher minimum soil temperatures. The large differences in climate we observed over small distances will likely lead to complex range shift dynamics and could buffer species from the negative effects of climate change.

## Introduction

Biologists have long recognized the fundamental role climate plays in determining the geographic distributions of species and biomes [1]–[3]. As a result, climate change is expected to induce shifts in the geographic ranges of species. This prediction is supported by the many observations of upward or poleward range shifts over the last 100 years consistent with observed warming [4] as well as range shifts inferred from the fossil record [5], [6]. Alarmingly, models of the impacts of future anthropogenic climate change on species ranges have forecasted widespread extinction risks as the climatic niche of many species disappears or shifts faster than species can likely migrate [7], [8].

However, these projections of climate change-induced range shifts (and subsequent extinction risks) are sensitive to the spatial scale at which the analyses are conducted [9]. Most range shift assessments rely on gridded maps of climate variables with grid cell sizes ranging from 800×800 m (e.g. PRISM [10] and WorldClim [11]) up to 50×50 km (e.g. [12]). The finer scale maps (800×800 m grid cells) capture a wide variety of climatic patterns, but the scales of these maps are still far coarser than the scales at which organisms experience their environment. Thus, these climate maps may hide fine-scale differences in climate that are important for organism distributions [13]. For example, north and south facing slopes separated by tens of meters may receive different amounts of solar radiation and experience very different temperature regimes [14], [15], which could lead to differences in species composition within these microhabitats.

The implication of significant fine-scale climatic heterogeneity that is not captured by coarse-scale climate maps is that projections based on these maps could fail to capture important range shift dynamics. For example, cool microhabitats (such as north-facing slopes in the Northern Hemisphere) near the contracting edge or core of a species’ distribution may allow populations of that species to persist if individuals can disperse to them from warmer microhabitats (such as south-facing slopes in the Northern Hemisphere), even if most of the surrounding landscape becomes unsuitably warm (as long as these microhabitat types comprise a total area large enough to support a population [16]). At the same time, warm microhabitats beyond the advancing edge of a species’ range may provide the first sites of colonization that allow that species to migrate to new locations. Thus, instead of needing to move hundreds of meters upward or hundreds of kilometers poleward to track suitable climate, many species may only need to move tens of meters from one microhabitat to another and could be buffered from the negative effects of climate change [17].

For such fine-scale climatic heterogeneity to strongly influence range dynamics, however, fine-scale differences in climate must be large relative to coarse-scale differences. We addressed this issue by examining the magnitude of fine-scale heterogeneity relative to coarse-scale heterogeneity in snow disappearance date and growing season soil temperature. Specifically, we deployed 284 microclimate sensors across a ∼1500 m elevation gradient spanning forest, subalpine and alpine biomes at Mount Rainier National Park. Our objectives were to 1) quantify snow disappearance date and soil temperature as a function of coarse-scale differences in elevation and exposure to storm tracks (i.e. being on the windward or leeward side of the mountain) and fine-scale differences in vegetation structure or topography, 2) compare fine-scale differences in climatic variables (that would be missed by climate models) to coarse-scale differences (that would be captured by climate models), and 3) determine whether fine-scale patterns in climatic variables related to topography (but not vegetation structure) are correlated with fine-scale patterns in vegetation characteristics. We focus on snow disappearance date and growing season soil temperature because snow disappearance date influences the length of the growing season (especially important in this region where the growing season can be very short due to the persistence of large winter snowpacks) while soil temperature strongly influences plant growth rates and other physiological processes [18]. These variables have also been shown to be strongly associated with patterns of distribution, abundance, productivity and diversity of plant species, at our sites and others [19]–[21]. Additionally, both variables are likely to change in the coming decades as a result of anthropogenic climate change, with rising temperatures and declining snowpacks leading to warmer and longer growing seasons [22], [23].

It has long been known that climate can vary dramatically at fine spatial scales (reviewed in [14] and [24]), but these patterns have only recently begun to be studied explicitly and systematically with respect to the impacts of climate change on species distributions. Studies have found that locations separated by only tens of meters experienced mean seasonal soil temperatures that differed by 3–7°C, equivalent to the average temperature difference experienced in locations separated by hundreds of meters in elevation or hundreds of kilometers in latitude [15], [21], [25]–[28]. Moreover, such large differences in temperature are known to strongly influence organismal performance [18], [29] and are greater than the expected increase in temperature due to climate change in many parts of the globe [30]. Similarly large differences were also observed in air temperature, snow cover duration or snow disappearance date over fine spatial scales in these studies. Our paper builds on these case studies and is notable for its large sample size of 284 sensors (important for assessing microclimate patterns in a statistically rigorous way), its explicit comparison of coarse- and fine-scale climatic heterogeneity (important for assessing the biases of coarse-scale models) and the broad environmental gradients covered (important for assessing how widespread these biases may be).

## Methods

### Ethics Statement

We obtained the necessary scientific research permits from Mount Rainier National Park, where all data collection occurred, before conducting this study. We did not sample any protected species.

### Study Area

**Figure 1:**
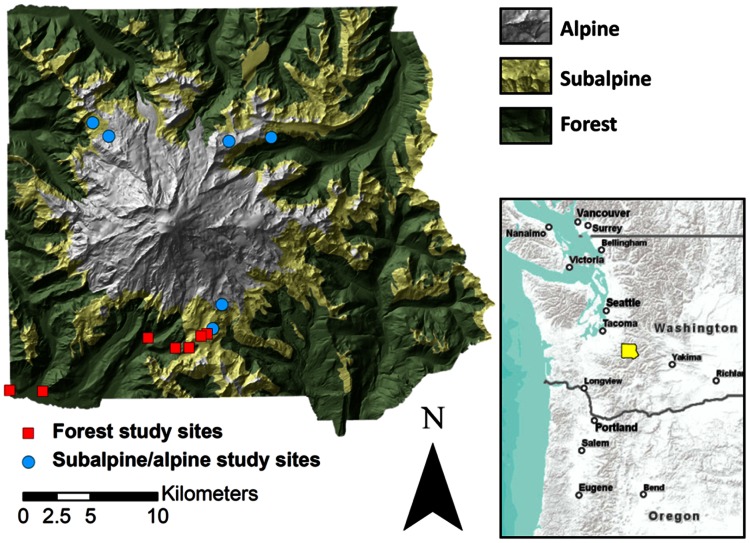
Study area. Mount Rainier National Park and its three major biomes, along with study site locations. Shading depicts topographic relief.

Mount Rainier National Park encompasses 95,354 ha of land in the western Cascade Mountains in Washington State, USA (Figure 1). The region experiences a temperate, maritime climate with mild, dry summers and cool, wet winters that produce large snowpacks. Elevation ranges from 518 m in the deep valley floors to 4392 m at the peak of Mount Rainier, the volcano located in the middle of the Park. The mountainous terrain produces steep climatic gradients: temperature decreases and precipitation increases with elevation, while the rainshadow effect produced by the volcano leads to lower precipitation on the east side of the Park. There are two primary climate stations in the Park. At the station located at 842 m elevation, mean annual temperature is 6.6°C and mean annual precipitation is 2030 mm; at the 1654 m station, mean annual temperature is 3.7°C and mean annual precipitation is 3005 mm (1981–2010 normals, NOAA National Climate Data Center – www.wrcc.dri.edu/Climsum.html).

The large climatic gradients create three major biomes in the Park. The forest biome extends from the lowest elevations of the Park up to about 1600–2000 m and is dominated by coniferous trees. The subalpine biome typically extends about 300 m above the upper limit of the forest and is a mosaic of conifer tree patches and subalpine meadows. The alpine biome occupies the highest elevations, stretching from 1900–2300 m to the summit of Mount Rainier, and consists of large patches of mostly continuous alpine meadows (dominated by forbs, grasses and dwarf shrubs) near the lower limit of the biome, with exposed rock, glaciers, bare soil, and cryptogams (mostly mosses, lichens, algae and cryptobiotic soil crusts) predominating above.

### Study Design

From September 2009 through October 2010, we deployed 284 soil temperature sensors (HOBO Pendants made by the Onset Computer Corporation and iButtons made by Maxim Integrated Products) across Mount Rainier at elevations ranging from 638 m to 2164 m as part of two different plant ecology studies where microclimate was measured as an explanatory variable. The first study took place in the forest biome and spanned the elevational range of forests on the south side of Mount Rainier. The second study took place in the subalpine and alpine biomes, with study sites set up at the lower limit of the subalpine biome and the upper limit of alpine meadows on three sides of the mountain (Figure 1). Microclimate data from these studies were ideally suited for our questions as they covered large elevational gradients with sensors at each location stratified by vegetation or topographic features expected to influence microclimate. Due to differences in study design (described below), we analyzed the microclimate data from the two studies separately. The sensors remained in place and logged data until we collected them in September/October 2010.

For each sensor, we calculated the values of four climatic variables: snow disappearance date, and average daily mean, maximum and minimum soil temperature. We could assess snow cover from soil temperature measurements because snow insulates soil from fluctuations in air temperature so that temperatures beneath the snowpack in this region remain constant around 0°C. Thus, the soil temperature data allowed us to determine whether snow was covering the sensor for each day the sensor was deployed using an algorithm that considers daily temperature ranges and maxima [31], [32]. We calculated average daily mean, maximum and minimum soil temperature for periods in Summer/Fall 2010 when all sensors in a study experienced snow-free conditions and reported data. This period was August 14 through October 3, 2010 for the sensors in the forest study and August 11 through August 18, 2010 for the sensors in the subalpine/alpine study. This meant we only used a portion of the snow-free temperature data for some sensors, even though temperatures outside this period are likely to also be ecologically relevant. However, it was necessary to use the same time period for all sensors in a study so that temporal differences in the snow-free period between locations did not confound our analysis of spatial differences in temperature.

**Figure 2:**
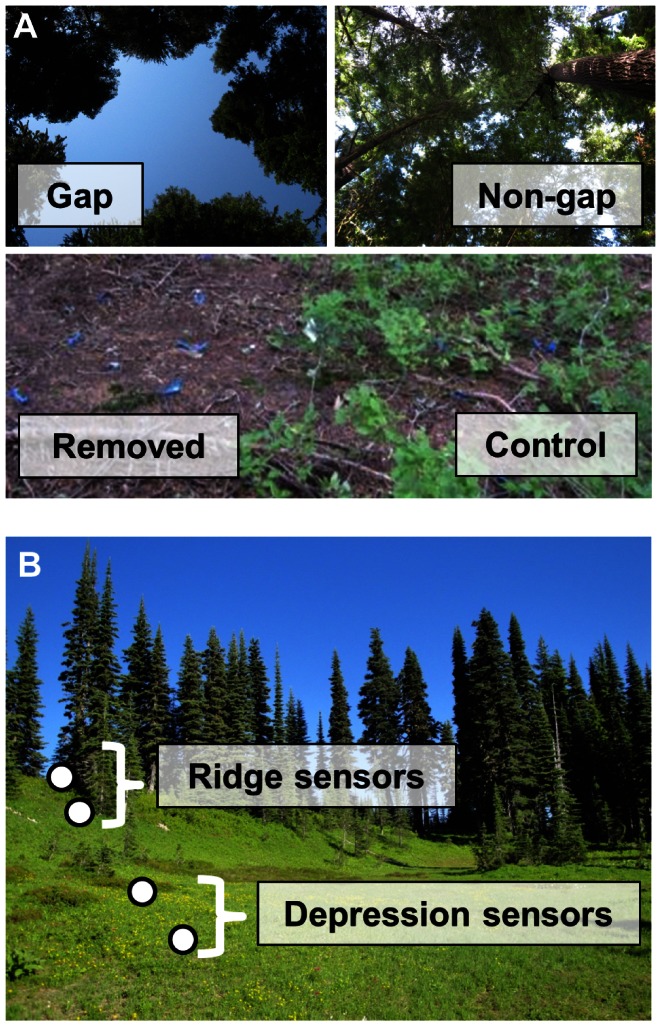
Temperature sensor deployment. Sensor deployment in (A) forest and (B) subalpine/alpine biomes. At each elevation in the forest biome (A), sensors were placed in gaps in the forest canopy (top left) and non-gaps with intact forest canopy (top right). Within each of these canopy types, sensors were located in plots where understory vegetation was removed (bottom left) and control plots where it was left undisturbed (bottom right). In the subalpine/alpine biomes (B), temperature sensors were located along transects running from depressions in the landscape to ridges.

Arrays of sensors were deployed at 13 sites throughout the Park. For the forest study, we established three study sites along an elevation gradient in Summer 2009, allowing us to calculate snow disappearance date in 2010. We quantified growing season soil temperature in Summer/Fall 2010 at these three sites plus an additional four sites along the same elevation gradient. Within each site, we deployed sensors under gaps in the forest canopy caused by tree falls (“gaps”) and in locations under intact canopy within 20 m of one of the gaps (“non-gaps”). Each study site contained five of these gap/non-gap pairs. Gaps were ∼130 m^2^ on average. Within each gap or non-gap location, we placed one sensor in a 5.5×1.5 m area where all understory vegetation up to 2 m tall had been experimentally removed since early Summer 2009 (the “removed” plot) and one sensor at an adjacent location 2 m away where the vegetation had been left undisturbed (the “control” plot) (Figure 2A, Table 1).

**Table 1:** Details of temperature sensor deployment.

Biome(s)	Time span ofdeployment	# sites	# sensorsper site	Total # sensors	Type of sensor	Sensor accuracy	Data logging interval	Sensor location
Forest	Summer 2009– Fall 2010	3	8	24	HOBO Pendants made by the Onset Computer Corporation	±0.53°C from 0° to 50°C	2 hours	Soil surface
Forest	Summer–Fall 2010	7*	20	140	HOBO Pendants made by the Onset Computer Corporation	±0.53°C from 0° to 50°C	1 hour	Soil surface
Subalpine/alpine	Summer 2009– Summer 2010	6	24	144	iButtons made by Maxim Integrated Products	±0.5°C from−10° to 65°C, or ±1°C from−30° to 70°C†	1, 2 or 4 hours†	3 cm below soil surface

* These sites include the three sites with sensors deployed in Summer 2009.

† Differences in accuracy and logging intervals were due to differences in the specific model of iButton sensor used.

For the subalpine/alpine study, we quantified microclimate at study sites on three sides of the mountain (south, northwest and northeast) which have different exposures to storm tracks and experience different precipitation regimes. On each side, we established two study sites, one close to the lower limit of the subalpine biome and one close to the upper limit of continuous alpine meadows, (below this limit, the ground is mostly vegetated while above it is almost entirely rock, glaciers and bare soil). These sites were about 200–300 m apart in elevation on each side of the mountain. Within each site, we established six linear transects that ran from a depression in the landscape up to a ridge (transects parallel to the slope) and were about 20 m in length. Within each transect, two sensors were located in the depression and two sensors were located on the ridge (Figure 2B, Table 1).

At each of the sensors in the subalpine/alpine biomes (where fine-scale sensor placement was stratified by topographic position and not vegetation structure), we measured vegetation characteristics in order to compare patterns in microclimate to ecological patterns. At the study sites near the lower limit of the subalpine biome, where closed-canopy forests transition to open meadows with increasing elevation, we measured percent cover by tree canopy above each sensor using a spherical densiometer (a gridded, hemisphere-shaped mirror used to estimate percent cover by foliage above a point on the ground), allowing us to assess the density of trees (higher values of tree canopy cover implies more or bigger trees). At the study sites near the upper limit of alpine meadows, where meadows transition to bare ground with increasing elevation, we estimated the percent of ground covered by vegetation at each sensor using a square-shaped PVC frame (1×1 m) placed on the ground adjacent to the sensor. String tied to the PVC frame created 100 evenly spaced grid points, allowing us to count the number of grid points overlaying vegetation in the area within the frame.

### Data Analysis

We used linear mixed effects models (LMMs) to characterize the relationships between the potential drivers of climate and each of the four climatic response variables [33]. The LMMs allowed us to estimate the effects of explanatory variables and their two-way interactions on the response variable (“fixed effects”) while statistically controlling for the effects of randomly selected experimental units on the response variable (“random effects”). At the forest sites, the drivers of climate were elevation, canopy structure (gap vs. non-gap) and understory structure (removed vs. control treatments), with gap/non-gap pair designated as a random effect. At the subalpine/alpine sites, the drivers of climate were side of the mountain (south vs. northwest vs. northeast), elevation (upper limit of alpine meadows vs. lower limit of the subalpine biome, treated as a categorical variable since there were only two values of elevation on each side of the mountain) and topographic position (depression vs. ridge), with sensor transect designated as a random effect. We verified that the residuals of these models were normally distributed, to validate our use of linear mixed effects models (rather than generalized linear mixed effects models).

For each model, we used Akaike’s information criterion (AIC) to select the most parsimonious combination of fixed and random effects to derive the “best-fit” model. Specifically, we used a three-step process following [33] where we (1) used AIC to determine the optimal random effects structure, selecting amongst several LMMs (fit with restricted maximum likelihood) that had different random effect terms (no random effects, random intercepts, random slopes or both), but the same fixed effect terms (which included all main effect and two-way interaction terms for each explanatory variable); (2) determined the optimal combination of fixed effect terms by using AIC to select amongst models (fit with maximum likelihood) with all possible combinations of fixed effect terms (but sharing the optimal random effects structure selected in the first step); and (3) fit a model with the random effects structure selected in the first step (which could be no random effects) and the fixed effects structure selected in the second step and considered this model to be our final “best-fit” model. This final model was fit with restricted maximum likelihood if it included random effects or maximum likelihood if it did not. All models were fit in R version 2.12.0 using the lme4 package for the LMMs [34], [35]. See Appendix S1 for more details of the model fitting and selection procedure.

We assessed the significance of the model coefficients using Markov chain Monte Carlo sampling implemented with the MCMCglmm package in R [36] or *t*-tests (when no random effects were included in the best-fit model). We then used the explanatory variable coefficients of the best-fit models to calculate the magnitude of differences in microclimate response variables relative to differences in the explanatory variables. For example, if the coefficients related to topographic position in the model of snow disappearance date at the subalpine/alpine sites indicated that the difference between ridges and depressions was 20 days, controlling for differences in other variables, then the effect of topographic position on snow disappearance date would be equal to 20 days. In order to compare the effects of elevation (which we consider a coarse-scale driver of climate) to the effects of other explanatory variables, we calculated the difference in snow disappearance date or temperature between two points 100 m apart in elevation for each model (controlling for differences in other variables). Like differences in climate amongst different sides of the mountain, differences in climate caused by a 100 m difference in elevation can typically be captured by coarse-scale climate models (e.g. PRISM), while differences caused by vegetation structure and fine-scale topography cannot. If one of the explanatory variables was not included in the best-fit model, we included the main effect of that variable in the final model for comparative purposes. This happened for one explanatory variable in one model (the understory structure variable in the snow disappearance date model in the forest study).

For sites in the subalpine/alpine biomes, we also fit linear models (LMs) to characterize the relationships between each of the four microclimate variables and percent cover by tree canopy at the lower elevation sites, and the relationships between each of the four microclimate variables and percent cover by ground vegetation at the higher elevation sites, for a total of eight LMs. In these models, the response variable was the vegetation characteristic while the explanatory variables were the microclimate variable, side of the mountain (included as a covariate) and their interaction. Using LMMs with sensor transect designated as a random effect did not improve model fit for any of the relationships, so we used the simpler LMs for all of these analyses. Next, we used AIC to select the best-fit LM. With one exception, this best-fit model included both the microclimate variable and side of the mountain, but not their interaction, as explanatory variables. In these cases, we used *t*-tests to assess the significance of the microclimate variable coefficient in the best-fit model in order to assess the significance of that microclimate variable. When modeling percent tree canopy cover at the lower elevation sites as a function of average daily minimum temperature and side of the mountain, neither explanatory variable nor their interaction was included in the best-fit model (i.e. the best-fit model was the null model with only an intercept). For this situation, we performed a *t*-test on the minimum temperature coefficient in a model that included both minimum temperature and side of the mountain, but not their interaction, as explanatory variables (i.e. a model with the same structure as the best-fit model for all the other vegetation-climate analyses) in order to assess the significance of minimum temperature.

We calculated the proportion of variance in the response variable explained by variance in the fixed effect explanatory variables (*r* ^2^) for all models, following [37].

## Results

**Figure 3:**
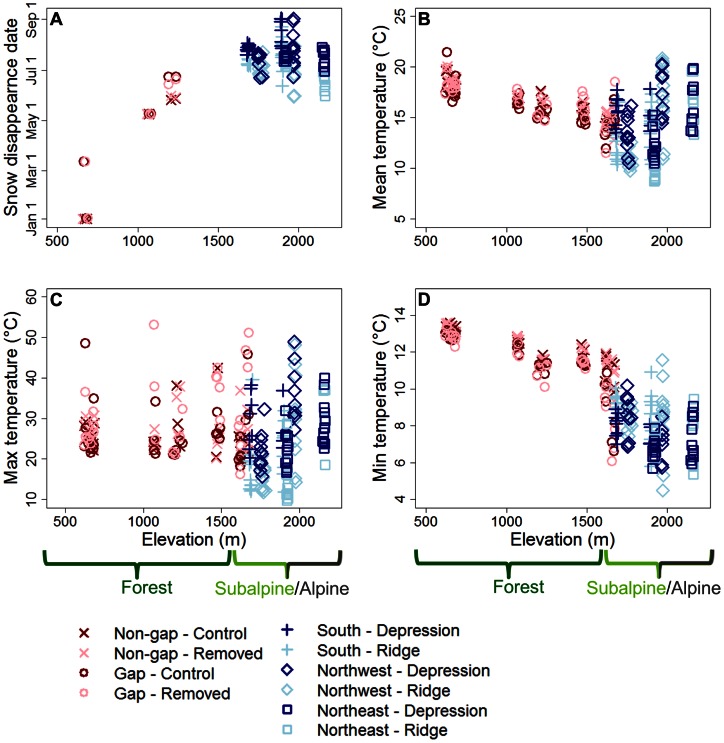
Patterns in climate. (A) Snow disappearance date in 2010 and average daily (B) mean, (C) maximum and (D) minimum soil temperature for a representative week during the growing season (August 11–18, 2010) plotted against elevation. Note the differences in scale on the axes showing temperature values. Points represent individual sensors with symbol type and color designating sampling stratification for forest (dark and light red) and subalpine/alpine sites (dark and light blue). “Non-gap”/“gap” refer to canopy structure categories while “control”/“removed” refer to understory structure categories (forest sites). “South”/“northwest”/“northeast” refer to sides of the mountain while “ridge”/“depression” refer to topographic positions (subalpine/alpine sites). Approximate biome ranges are shown below the elevation axes.

**Figure 4:**
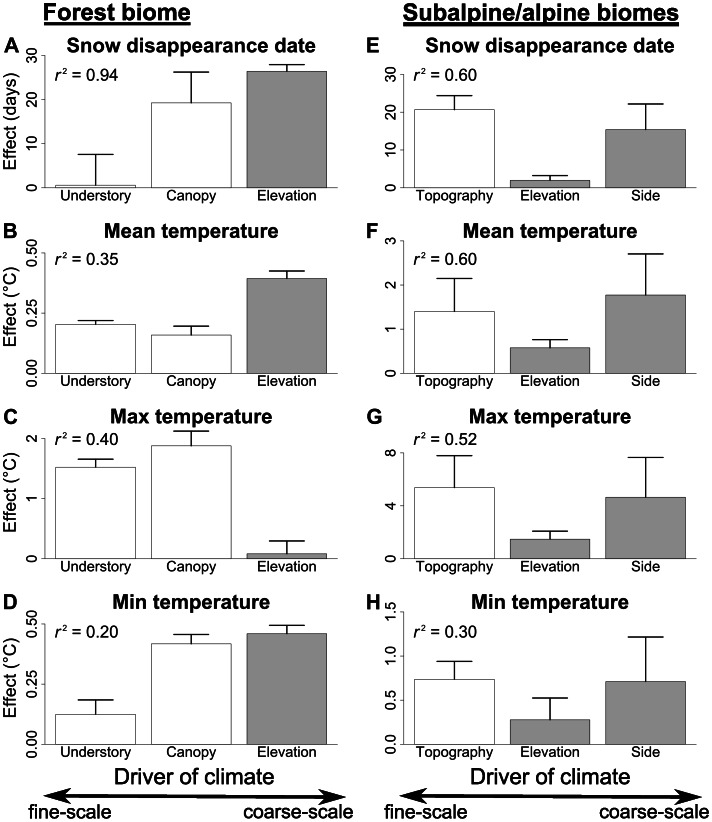
Effects of fine- and coarse-scale drivers of climate. The effects of fine- and coarse-scale drivers of climate on snow disappearance date and the average daily values of mean, maximum and minimum growing season soil temperature. Bars show differences in snow disappearance date or temperature attributed to the effect of different drivers of climate by the best-fit model, with standard error. The effect of elevation was standardized to the effect of a 100 m difference in elevation. Bars filled with gray represent drivers that are coarse enough in scale to be captured by typical climate models (>1 km) while unfilled bars represent drivers too fine in scale to be captured by these models (≤20 m). Fine-scale drivers of climate often had a greater effect on snow or soil temperature than coarse-scale drivers.

Variations in climate were explained by both coarse- and fine-scale drivers, with best-fit models having *r* ^2^ values ranging between 0.20 and 0.94 (Table 2). As expected, higher elevations experienced later snow disappearance dates and lower average daily mean and minimum temperatures (Figure 3A, B, D). However, the relationship between elevation and average daily maximum temperature was weak, and variability in this parameter was dominated by variability amongst locations at similar elevations (Figure 3C). At the subalpine/alpine sites, snow disappearance date and temperature varied depending on what side of the mountain sensors were on – e.g. the south side experienced later snow disappearance dates on average, probably because this side of the mountain is the most exposed to winter storms and receives the largest amount of winter precipitation. However, there were also substantial differences amongst locations at similar elevations for each of these variables that could be attributed to vegetation structure or topographic position (Figures 3, 4). We also assessed heterogeneity in growing degree days (GDD – calculated as the sum of daily mean soil temperatures for all days where the daily mean soil temperature was over 5°C), which showed patterns very similar to those of snow disappearance date (results not shown due to limitations of the data – sensors were not deployed long enough to estimate GDD for the full year or growing season, which could bias comparisons of GDD amongst locations).

**Table 2:** Best-fit models for the climatic response variables.

Study	Climatic response variable	Model formula*	*r* ^2^
Forest: stratification by vegetation structure	Snow disappearance date	SDD = *f* { **elev**+**canopy**+understory†+ (1|pair) }	0.94
Forest: stratification by vegetation structure	Average daily mean temperature	Tmean = *f* { **elev**+canopy+understory+elev:canopy+**elev:understory**+**canopy:understory**+(0+canopy|pair) }	0.35
Forest: stratification by vegetation structure	Average daily maximum temperature	Tmax = *f* { elev+**canopy**+understory+**elev:canopy**+**elev:understory**+**canopy:understory**+(0+canopy|pair) }	0.40
Forest: stratification by vegetation structure	Average daily minimum temperature	Tmin = *f* { **elev**+canopy+**understory**+**elev:canopy**+**canopy:understory**+(0+canopy|pair) }	0.20
Subalpine/alpine: stratification by topographic position	Snow disappearance date	SDD = *f* { **side**+**elev**+**topo**+**elev:topo**+**side:elev**+(1|tran) }	0.60
Subalpine/alpine: stratification by topographic position	Average daily mean temperature	Tmean = *f* { **side**+elev+topo+**elev:topo**+**side:elev** }	0.60
Subalpine/alpine: stratification by topographic position	Average daily maximum temperature	Tmax = *f* { **side**+elev+topo+**elev:topo**+**side:elev** }	0.52
Subalpine/alpine: stratification by topographic position	Average daily minimum temperature	Tmin = *f* { side+elev+**topo**+side:elev }	0.30

* Parameters in bold have significant coefficients (*p*<0.05). For the forest biome study, elev = elevation; canopy = forest canopy. structure, gap or non-gap; understory = understory vegetation structure, removed or control; pair = gap/non-gap pairings. For the subalpine/alpine biomes study, side = side of the mountain, south or northwest or northeast; elev = elevation; topo = topographic position, ridge or depression; tran = sensor deployment transect. The colon indicates an interaction effect between two explanatory variables. The parentheses indicate the term is a random effect – all other terms are fixed effects. If *fe* is a particular fixed effect and *re* is a particular random effect, then (1|*re*) indicates the intercept was allowed to vary randomly with respect to *re* while (0+*fe*|*re*) indicates the interaction of *fe* and *re* was allowed to vary randomly. The case of both the intercept and interaction being allowed to vary randomly was not included in any of the best-fit models.

† Understory was not included in the best-fit model, based on AIC, but was retained for comparative purposes.

### Forest Biome: Stratification by Vegetation Structure at Fine Scales

Snow disappearance date was later at higher elevations and in canopy gaps, while understory vegetation structure had little effect (Figure 3A). We also found that the effect of fine-scale differences in canopy structure (gaps vs. non-gaps) on snow disappearance date was similar to the effects of coarse-scale differences in elevation (100 m elevation differences) (Figure 4A). Thus, snow disappearance date differed as much at fine scales (where locations differed in forest canopy structure) as it did over coarse spatial scales.

As expected, growing season soil temperature generally declined with increasing elevation. Canopy gaps had higher maximum temperatures, but lower minimum and mean temperatures relative to non-gaps (Figure 3B–D). Canopy structure had a similar or greater effect on temperature than a 100 m change in elevation for average daily maximum and minimum temperature (Figure 4C, D). Locations where understory vegetation was removed experienced higher maximum and mean temperatures, but lower minimum temperatures, relative to control plots where vegetation was undisturbed (Figure 3B–D). Understory structure had a greater effect on average daily maximum temperature than a 100 m change in elevation, but had weaker effects on average daily mean and minimum temperature (Figure 4B–D). Overall, there was about as much heterogeneity in temperature at fine scales (differing vegetation structure) as there was at coarse scales (100 m differences in elevation).

### Subalpine/alpine Biomes: Stratification by Topographic Position at Fine Scales

Snow disappearance date was later on the south side of the mountain, at higher elevations and in topographic depressions (Figure 3A). Furthermore, the effect of fine-scale topographic differences (depressions vs. ridges) on snow disappearance date was similar to the effects of coarse-scale differences in elevation (100 m difference in elevation) and side of the mountain (Figure 4E). In other words, snow disappearance date differed as much over fine spatial scales as it did over coarse spatial scales.

Growing season soil temperatures during our sampling period were lower on the northeast side of the mountain than on the northwest and south sides, potentially because the meadows are at higher elevations on this side of the mountain. On a given side of the mountain, higher elevations (the upper limit of alpine meadows) had higher mean and maximum temperatures, but lower minimum temperatures, than lower elevations (the lower limit of the subalpine biome). Compared to ridges, depressions had higher mean and maximum temperatures but lower minimum temperatures (Figure 3B–D). We found that the effects of fine-scale topographic differences (depressions vs. ridges) were similar to the effects of coarse-scale differences in elevation and side of the mountain for average daily mean, maximum and minimum temperature (Figure 4F–H). Overall, there was as much heterogeneity in temperature at fine scales as there was at coarse scales.

**Figure 5:**
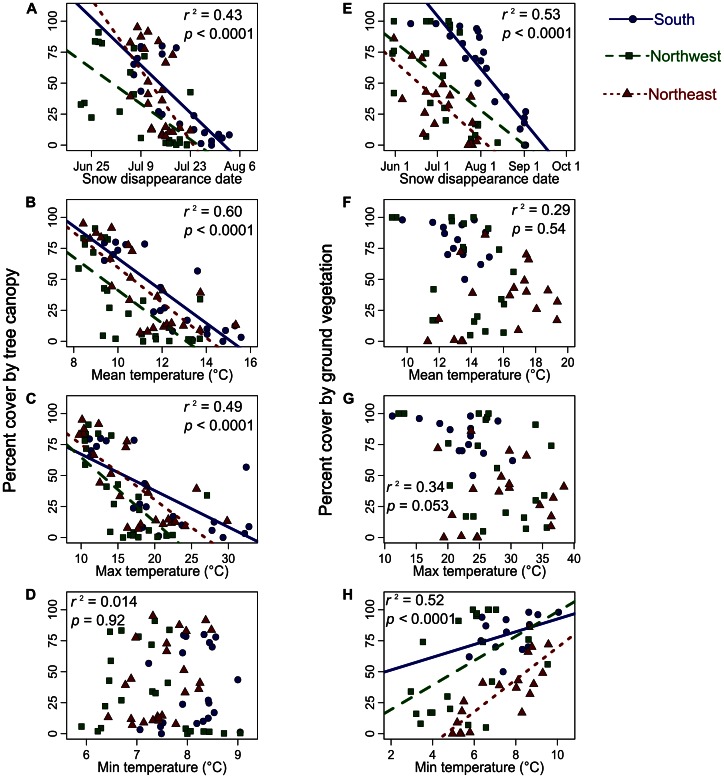
Relationships between vegetation characteristics and microclimate. (A–D) Percent cover by tree canopy at sites near the lower limit of the subalpine biome and (E–H) percent cover by ground vegetation at sites near the upper limit of alpine meadows plotted against the four microclimate variables (snow disappearance date and average daily mean, maximum and minimum soil temperature) on each of the three sides of the mountain. The *r* ^2^ values are for models that included the microclimate variable and side of the mountain as explanatory variables, while the *p* values indicate the significance of the microclimate variable in these models. Regression lines are shown for significant *p* values (<0.05).

Several microclimate variables were significantly correlated with vegetation characteristics (Figure 5). At study sites near the lower limit of the subalpine biome, percent cover by tree canopy was lower where snow disappearance date was later and average daily mean and maximum soil temperatures were higher (*p*<0.0001). At study sites near the upper limit of alpine meadows, percent cover by ground vegetation was lower where snow disappearance date was later and average daily minimum soil temperature was lower (*p*<0.0001).

## Discussion

Our study suggests that climatic heterogeneity at the fine spatial scales most organisms experience their environment is substantial, implying that projections based on coarse-scale climate models will not capture the full complexity of range shifts in response to climate change. Specifically, we found large differences in snow disappearance date and growing season soil temperatures over small distances (Figures 3, 4), differences that were sometimes as large as those experienced when travelling hundreds of meters upward in elevation or several kilometers to a different side of the mountain. These microclimate variables have been shown to be strongly associated with plant species distributions and abundances [20], [21], suggesting that the microclimate heterogeneity we observed is important for plant communities. We also found that vegetation characteristics (canopy and ground vegetation cover) can be strongly correlated with the microclimate variables influenced by fine-scale topographic features (Figure 5), further suggesting that the fine-scale climatic heterogeneity we observed is ecologically important. However, because we did not measure species distributions or abundances in this study, we cannot conclusively state that the microclimate heterogeneity we observed is linked to species distributions or abundances at Mount Rainier. Nonetheless, understanding fine-scale climatic heterogeneity will likely be critical for management, as cool or snowy microhabitats could provide an important buffer against the negative effects of climate change on biodiversity. Thus, when assessing potential species range shifts in response to climate change, it is critical for ecologists to consider fine-scale patterns in climate in addition to other important factors such as broad-scale climate patterns, dispersal constraints, biotic interactions and evolutionary dynamics.

### Explanations of Fine-scale Climatic Heterogeneity

In the forest biome, a complex interplay between elevation and vegetation structure is likely responsible for the heterogeneous patterns in snow disappearance date and soil temperature we observed. For example, locations under tree canopy gaps likely experienced later snow disappearance dates than locations under an intact canopy (Figure 3A) because tree canopies intercept snowfall where it can rapidly sublimate or melt instead of being incorporated into the snowpack on the ground [38]. Tree canopies also increase incoming longwave radiation (which increases ablation rates) and this effect can sometimes be greater than the effect of canopies decreasing incoming shortwave radiation by shading the snowpack (which reduces ablation rates), leading to a net effect of canopies increasing ablation rates [39]. Although the presence of trees has also been shown to lead to longer snow persistence by shading the snowpack and decreasing wind speeds (reducing incoming sensible and latent heat fluxes) [38], these effects appear to be relatively weak at our study sites. Increased shading from tree canopies and understory vegetation in forest locations probably led to substantially lower maximum soil temperatures (Figure 3C). But these low sky exposure locations also experienced higher minimum soil temperatures (Figure 3D), probably due to vegetation emitting more longwave radiation (which warms the surface) than the night sky [14]. Differences in mean soil temperatures appeared to be the net effect of these two counteracting influences of sky exposure, with mean soil temperatures being higher in the shadier non-gap locations, but lower in the shadier undisturbed understory vegetation locations (Figure 3B).

Similarly, in the subalpine/alpine biomes we found that both coarse- and fine-scale features had large effects on climate. For example, snow disappeared substantially later from depressions in the landscape than from ridges only ∼20 m away, likely because snow typically collects in these depressions while it is blown off of ridges and because shading from surrounding slopes can reduce ablation rates [24]. Feedbacks between vegetation and climate are also likely to influence fine-scale climatic variability. At the lower elevation sites, for example, patches of trees with trunks sticking out above the snowpack emit substantial amounts of longwave radiation which quickens the ablation of snow next to the tree patch and can lead to earlier snow disappearance dates. Trees can also intercept snowfall, reducing snowpack accumulation under canopy and resulting in earlier snow disappearance [38]. These effects can lead to a positive feedback, where trees establish in microsites with earlier snow disappearance dates (e.g. ridges), and the established trees lead to even earlier snow disappearance dates and more tree establishment. This result is consistent with previous studies from subalpine meadows in the region that have documented increased tree establishment on ridges that tend to have earlier snow disappearance dates [40], [41].

A striking pattern to emerge from our data was that mean and maximum soil temperatures were greater at higher elevations within the subalpine/alpine biomes (though minimum soil temperatures were lower). Feedbacks between climate and vegetation likely play important roles in producing this temperature pattern. First, tree cover declines with increasing elevation, leading to less shading and potentially higher daytime soil temperatures, especially during the sunny growing season when our data were collected. This explanation is supported by the negative correlation we observed between percent canopy cover (a measure of tree density) and mean/maximum soil temperature in the subalpine/alpine biomes (Figure 5B, C). Second, ground vegetation density declines with increasing elevation, which can lead to lower organic matter content in the soil and lower soil moisture levels. The lower moisture levels probably cause the soil to have a lower heat capacity, leading to greater temperature change per unit of energy input and hence higher maximum temperatures and lower minimum temperatures. This second explanation is supported by the pattern of low soil organic matter content and soil water holding capacity at high elevations in Mount Rainier’s subalpine/alpine biomes (Appendix S2). Soil characteristics also have important effects on vegetation in subalpine/alpine environments [42], creating the possibility for complex feedbacks amongst soil, vegetation and climate. These two hypotheses are not mutually exclusive, and further study is needed to assess the importance of each. Regardless, our results suggest that even if patterns in climate are ultimately responsible for patterns in vegetation, the feedback effect of vegetation on soil temperature (either directly, or indirectly through the effects of vegetation on soil characteristics which then affect temperature) appears to at times be stronger than the original forcing of physiographic effects on soil temperature.

### Implications of Fine-scale Climatic Heterogeneity for Species Distributions in a Warming World

Since snow disappearance date and growing season temperature vary dramatically over short distances, species whose distributions are primarily constrained by these climate variables may not need to migrate long distances to remain in suitable habitat even when there are large changes in climate. For example, in the subalpine/alpine biomes we found that the average difference in snow disappearance date between depressions and ridges separated by only ∼20 m was often one month or more. This is an especially large difference considering the ground is typically only free of snow for 3–5 months out of the year at these elevations. Snow manipulation experiments have shown that differences of this magnitude have large impacts on the phenology, species composition, diversity and productivity of plant communities [20]. Thus, these snowy microhabitats have the potential to serve as refugia for species in a warmer world and provide linkages to new areas of suitable climate, implying that fine-scale climatic heterogeneity could buffer species from climate change [27], [43], [44], as it may have done during past periods of rapid climate change [45]. Given that we did not stratify our sensors along all gradients likely to produce fine-scale differences in climate (e.g. wind direction, aspect – [14], [24]), our results may even be an underestimate of the magnitude of fine-scale heterogeneity.

The importance of topographic heterogeneity for creating climatic heterogeneity shown in this study and others [15], [21], [25]–[28] also suggests that mountainous regions will be important for providing climatic refugia in a warming world. However, mountain biotas will still likely face unique challenges. For example, organisms currently living on or near summits will not be able to shift upwards to track suitable climate, and deep valleys between mountains will likely pose serious obstacles to poleward shifts [7]. Broad-scale modeling will continue to be important for addressing these problems. Furthermore, fine-scale environmental heterogeneity does not guarantee that biodiversity will be buffered from climate change. It is possible for heterogeneity to produce small, isolated patches of habitat that cannot support many species, producing a negative effect on diversity [16]. Thus, whether the net effect of heterogeneity on maintaining diversity will be positive during a period of rapid climate change remains an open question.

Different kinds of cool or snowy microhabitats will likely differ in their effectiveness as refugia in a warming world. First, the abundance of microhabitat types will strongly influence how effective they can be as refugia. For example, depressions in the landscape in the subalpine/alpine biomes may have a high likelihood of serving as refugia because they are a common topographic feature. Second, the longevity of microhabitat types will affect their ability to act as refugia. For example, canopy gaps may disappear relatively quickly as trees establish in them, forcing species that might use gaps as refugia to migrate amongst gaps, which may not be possible for some species (though others may be adapted to this migration). In contrast, depressions in the landscape could provide more long-term refugia. Third, the temporal climatic heterogeneity experienced in microhabitat types may affect how well they can serve as refugia. For example, gaps had lower daily minimum and higher daily maximum temperatures compared to non-gaps, showing that these microhabitats experience a wide variety of temperatures. This heterogeneity may favor some species but not others. A final complicating factor influencing how and whether microhabitat types can serve as climatic refugia are the non-climatic conditions associated with them. For example, depressions may differ from other topographic positions in soil characteristics, which could prevent some species from using them as snowy microrefugia.

An important caveat to these findings is that they are based on one year of data. Spatial patterns in climate can change from year to year due to differences in prevailing synoptic weather patterns [46]–[48], so the patterns we observed in the year we conducted this study may not represent typical spatial patterns. However, the year of our study was a fairly typical year in terms of snow disappearance date and in terms of growing season air temperature for the past few decades (Appendix S3). And although spatial patterns in climate can vary year to year, the patterns are generally constant from one year to the next, especially in terms of snow [49]–[52]. For example, locations with later snow disappearance dates in one year tend to have later snow disappearance dates in other years, even though the spatially averaged snow disappearance date varies from year to year.

### Challenges and Opportunities for Management

To best protect biodiversity in a period of rapid climate change, conservation biologists and resource managers will require realistic assessments of future species distributions [53]. Thus, incorporating fine-scale climatic heterogeneity is essential for improving projections of species range shifts and extinction risks. Current coarse-scale models of the relationships between climate and species distributions ignore fine-scale heterogeneity and may therefore overestimate the distance species must migrate to track suitable climate (because forecasted range shifts are necessarily at the resolution of the model), and overpredict habitat loss and extinction risks ([54]–[57], but see [58]). Ecologists have previously criticized these bioclimate envelope models for only predicting where the climate that is currently associated with a species distribution will shift to and failing to account for biotic factors that could affect a species’ ability to track these climate shifts (dispersal limitations, biotic interactions and evolutionary changes) [59]. However, the limitation of model spatial resolution could undermine predictions not only of species’ abilities to track shifts in climate but also of the climate shifts themselves.

In addition to more realistically forecasting range shifts, knowledge of fine-scale climatic heterogeneity may also allow managers to increase species and ecosystem resilience to climate change. For example, protecting microhabitats with cooler temperatures or later snow disappearance dates could become increasingly important as climate change occurs because these microhabitats may provide critical refugia for species. Additionally, our results suggest that planting seedlings and sowing seeds at a variety of microhabitats when restoring degraded sites is an important bet-hedging strategy because it could increase the probability that species establish in microsites that remain suitable as climate change progresses, even if those microsites are only marginally suitable now. Thus, information on fine-scale climate heterogeneity has the potential to be useful for natural area protection and restoration when taken together with other important factors such as edaphic constraints, biotic interactions, genetic diversity and financial costs [60]. More detailed and longer term studies are needed to assess whether microclimate heterogeneity can contribute substantially to plant establishment and restoration efforts in a warming world.

### Conclusions

We have shown that snow disappearance date and growing season soil temperature vary dramatically over small distances due to differences in vegetation structure and topography. In fact, fine-scale features such as gaps in the forest canopy or small depressions in the landscape can produce differences in snow disappearance date and soil temperature as large as those produced by shifting hundreds of meters up a mountain slope. This large degree of fine-scale spatial heterogeneity may provide an important buffer against the negative effects of rapid climate change, as many species may only need to migrate tens of meters from one microhabitat to another in order to track suitable climate, as opposed to shifting hundreds of meters upward in elevation or hundreds of kilometers poleward. Climate change will undoubtedly pose serious threats to biodiversity, but knowledge of fine-scale climatic heterogeneity may allow managers to better assess and potentially alleviate some of these threats.

## Supporting Information

Appendix S1 **Model fitting and selection procedure.**
(PDF)

Appendix S2 **Soil characteristics in the subalpine and alpine biomes.**
(PDF)

Appendix S3 **Climatological context of the study.**
(PDF)
