# Supplementary material for: Spatial Heterogeneity in Ecologically Important Climate Variables at Coarse and Fine Scales in a High-Snow Mountain Landscape
Source: PLoS One. 2013 Jun 7;8(6):e65008. doi: 10.1371/journal.pone.0065008 (PMC3676384; doi:10.1371/journal.pone.0065008)
Supplement: Appendix S1 — Model fitting and selection procedure. (PDF) [file pone.0065008.s001.pdf]

Appendix S1: Model fitting and selection procedure

We used linear mixed effects models (LMMs) with gap/non-gap pair (for the forest biome) or transect (for the subalpine/alpine biomes) designated as a random effect to characterize the relationships between the explanatory variables and the four climatic response variables (snow disappearance date and average daily mean, maximum and minimum growing season soil temperature). For each climatic variable in either the forest or subalpine/alpine biomes, we used a three-step process to select the "best-fit" combination of random and fixed effects for the model following [1].

First, we determined the optimal random effects structure by using restricted maximum likelihood estimation to fit several LMMs which included main effect terms for all explanatory variables and terms for all two-way interactions, as well as different combinations of random effect terms (random intercepts only, random slopes only, or both random intercepts and random slopes). Then we calculated the Akaike's information criterion (AIC) value for each LMM to identify the best-fitting model by choosing the model with the lowest AIC or the model with the fewest parameters when AIC values of the lowest AIC model and other candidate models differed by less than 2 AIC units [2]. If the estimated variance explained by the random effects was zero, we used a model with only fixed effects, i.e. a linear model (LM).

Second, we determined the optimal fixed effects structure by creating models with all possible combinations of fixed effect terms (fit using maximum likelihood estimation) but sharing the same optimal random effects structure selected in the first step (which could be no random effects). We then calculated the AIC value for each model and selected the model with the lowest AIC or the model with the fewest parameters when AIC values of the lowest AIC model and other candidate models differed by less than 2 AIC units [2].

Third, we fit a model using restricted maximum likelihood estimation for LMMs and maximum likelihood estimation for LMs with the random effects structure selected in the first step (which could be no random effects) and the fixed effects structure selected in the second step. We considered this model to be our final "best-fit" model. All models were fit in R version 2.12.0 (R Development Core Team 2010) and the LMMs were fit using the lme4 package [3,4].

References

1. Zuur AF, Ieno EN, Walker NJ, Saveliev AA, Smith GM (2009) Mixed effects models and extensions in ecology with R. New York: Springer. 574 p.

2. Burnham KP, Anderson DR (2002) Model selection and multimodel inference: A practical information-theoretic approach. New York: Springer. 488 p.

3. R Development Core Team (2010) R: A language and environment for statistical computing.

4. Bates D, Maechler M, Bolker B (2011) lme4: Linear mixed-effects models using S4 classes. R package version 0.999375-39.
