# Supplementary material for: Spatial Heterogeneity in Ecologically Important Climate Variables at Coarse and Fine Scales in a High-Snow Mountain Landscape
Source: PLoS One. 2013 Jun 7;8(6):e65008. doi: 10.1371/journal.pone.0065008 (PMC3676384; doi:10.1371/journal.pone.0065008)
Supplement: Appendix S2 — Soil characteristics in the subalpine and alpine biomes. (PDF) [file pone.0065008.s002.pdf]

Appendix S2: Soil characteristics in the subalpine and alpine biomes

To characterize spatial patterns in soil organic matter content and water holding capacity in the subalpine and alpine biomes, we collected soil samples from seven plots along an elevation transect from the upper limit of the forest biome up to the lower limit of the alpine biome on the south side of Mount Rainer. Within each plot, we sampled from six locations differing in topographic position – two ridges, two depressions and two slopes – for a total of 42 samples for both organic matter content and water holding capacity tests.

To calculate soil organic matter content (the proportion of organic material in the soil), we first dried the sample to remove all moisture, recorded the mass of the dry soil, placed the dried soil in a muffle furnace set to 400°C for 10 hours to volatilize all organic matter and then recorded the mass of the remaining unburned soil. We subtracted the mass of the unburned soil from the mass of the dried soil to obtain the mass of the organic matter in the sample. We then calculated organic matter content as the mass of organic matter in the sample divided by the mass of the dried soil sample.

To calculate soil water holding capacity, we dried each sample to remove all moisture, recorded the mass of the dry soil, saturated the soil with water and then recorded the mass of the wet soil. We subtracted the mass of the dry soil from the mass of the wet soil to obtain the mass of the water held by the soil. We then calculated soil water holding capacity as the mass of the water held by the soil divided by the mass of the dry soil.

We used generalized linear models to test the importance of elevation, topographic position and their interaction on organic matter content (Beta error distribution) and soil water holding capacity (Gamma error distribution). The best-fit model for both soil variables included elevation but not topographic position or the interaction of elevation and topographic position (based on AIC – Akaike's information criterion). To assess the significance of pairwise differences in organic matter content and soil water holding capacity amongst elevations, we performed multiple comparison tests in R version 2.12.0 [1] using the multcomp package [2]. Both organic matter content and soil water holding capacity are lower at higher elevations and seem to cross a threshold around 1900 m, the upper ecotone of the subalpine biome (Figure S2-1). Below 1900 m the ground is mostly covered in vegetation, while above 1900 m it is mostly bare.

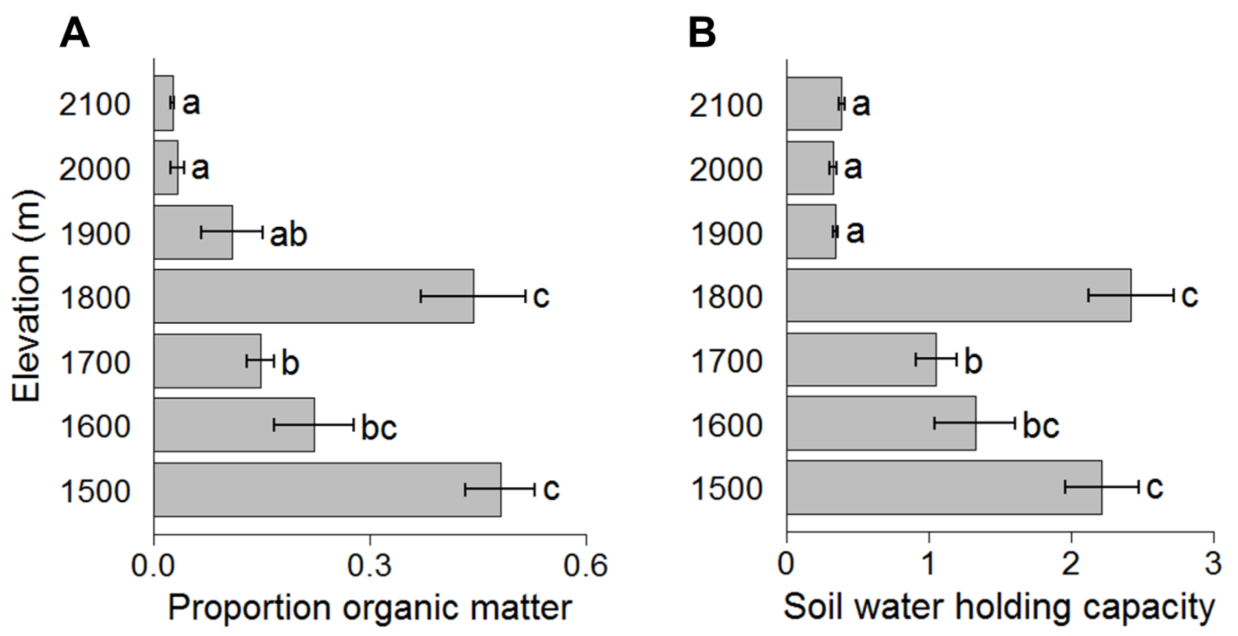

Figure S2-1. Mean soil characteristics across elevation. Bars with same letter are not significantly different (multiple comparison test, $\alpha = 0.05$). Error bars show standard error.

References

1. R Development Core Team (2010) R: A language and environment for statistical computing.

2. Hothorn T, Bretz F, Westfall P (2008) Simultaneous inference in general parametric models. Biometrical Journal 50: 346-363.
