# Supplementary material for: Spatial Heterogeneity in Ecologically Important Climate Variables at Coarse and Fine Scales in a High-Snow Mountain Landscape
Source: PLoS One. 2013 Jun 7;8(6):e65008. doi: 10.1371/journal.pone.0065008 (PMC3676384; doi:10.1371/journal.pone.0065008)
Supplement: Appendix S3 — Climatological context of the study. (PDF) [file pone.0065008.s003.pdf]

Appendix S3: Climatological context of the study

Data from a climate station located in Mount Rainier National Park at 1564 m elevation (the Paradise SNOTEL site, www.wcc.nrcs.usda.gov/snow) indicate that 2010, the year microclimate data were collected for this study, was a fairly typical year in terms of snow disappearance date and growing season air temperature for the past few decades. Snow disappearance date at this station for 2010 was within one standard deviation of the average snow disappearance date for 1984-2010 (Figure S3-1). Average daily mean, maximum and minimum air temperatures for the periods we analyzed soil temperatures in 2010 were all within one standard deviation of the 1984-2010 averages, with the single exception of average daily maximum temperature for the forest biome sampling period, which was 1.8°C lower than average. If typical values of climate variables at the climate station are associated with typical spatial patterns of climate variables in the Park, the patterns we observed in 2010 are likely representative of what typically occurs.

To further assess whether spatial patterns in snow disappearance date might vary year to year in the Pacific Northwest, we assessed spatial patterns in snow disappearance dates at snow monitoring stations in the region (SNOTEL sites). We found that spatial patterns in snow disappearance data in 2010 were not qualitatively different from typical years (the 1982-2011 climatology), or from low or high snow years (Figure S3-2). These spatial patterns are on a much coarser scale than our study, but are consistent with the findings of other fine-scale studies that spatial patterns in snow disappearance dates do not differ greatly year to year [1-4].

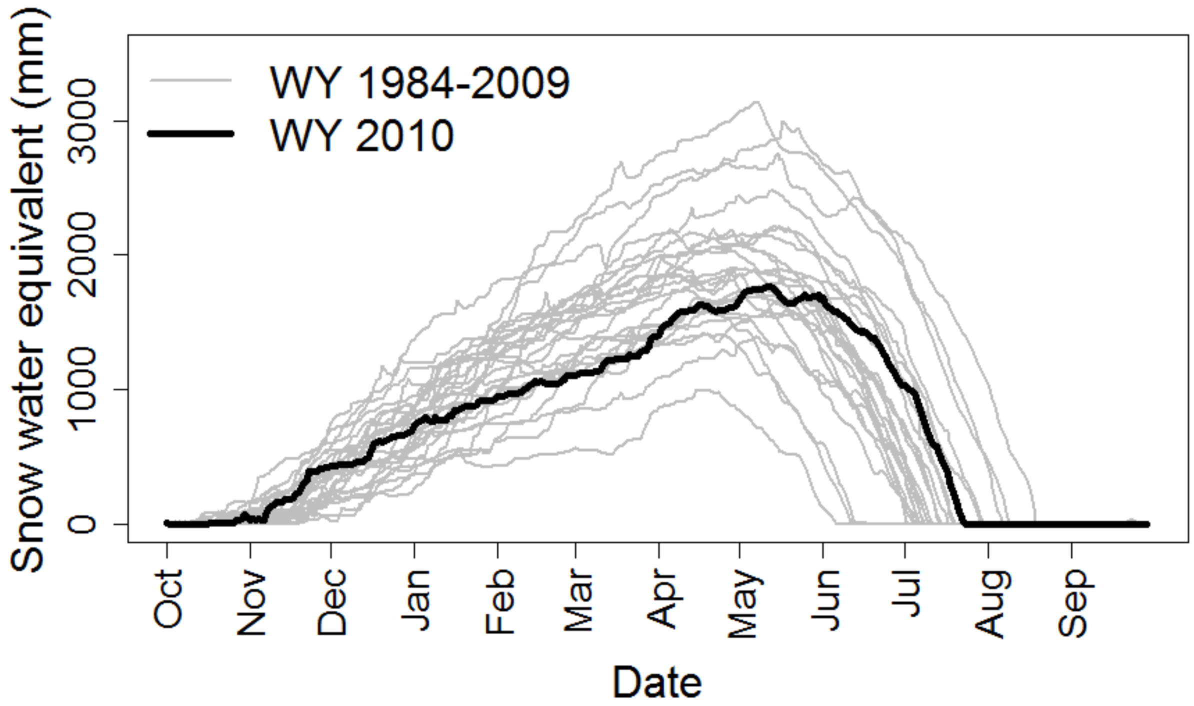

Figure S3-1. Year-to-year variation in snow at Mount Rainier. Data are from the Paradise SNOTEL site. Snow water equivalent is the amount of water contained in the snowpack, and represents the depth of water that would result if the snow melted. The years are water years, which run from October 1 of the previous year to September 30 of the given year (e.g. water year 2010 ran from October 1, 2009 to September 30, 2010).

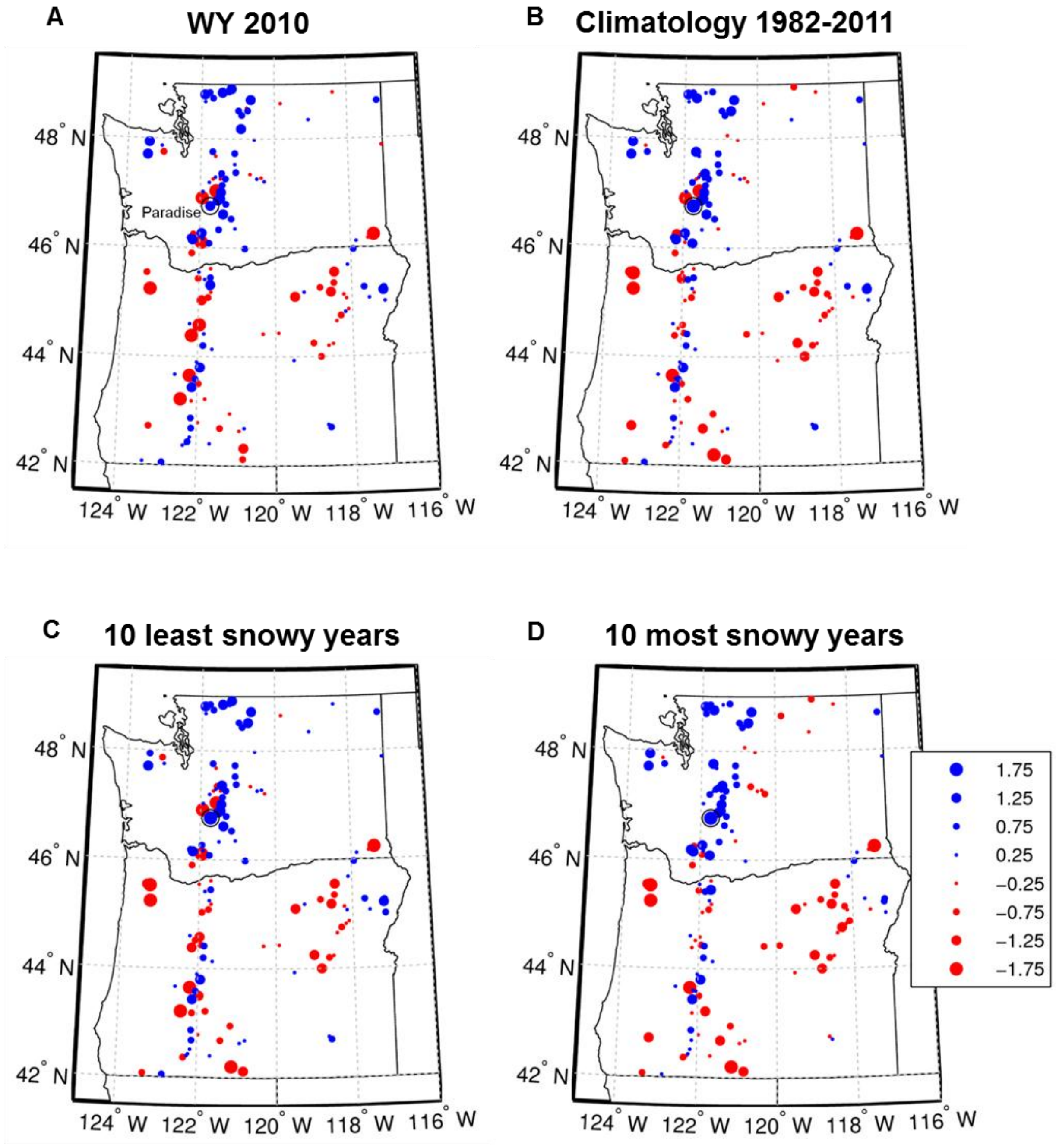

Figure S3-2. Spatial patterns in snow disappearance dates over time in Washington and Oregon. The dots represent snow monitoring stations (SNOTEL sites), with the station nearest to the study sites circled (Paradise). The size and color of the dots indicate how many standard deviations each station's snow disappearance date is from the mean snow disappearance date across all sites for the given time period, with blue representing later than average snow disappearance and red representing earlier. (A) Spatial patterns of snow disappearance date in 2010, (B) the average snow disappearance date patterns for 1982-2011, (C) the average snow disappearance date patterns for the 10 most snowy and (D) 10 least snowy years in the 1982-2011 period, with most and least snowy being based on the ranked peak snow accumulation value.

References

1. Erickson T, Williams M, Winstral A (2005) Persistence of topographic controls on the spatial distribution of snow in rugged mountain terrain, Colorado, United States. Water Resour Res 41: W04014. doi: 10.1029/2003WR002973.

2. Deems JS, Fassnacht SR, Elder KJ (2008) Interannual consistency in fractal snow depth patterns at two Colorado mountain sites. J Hydrometeorol 9: 977-988. doi: 10.1175/2008JHM901.1.

3. Sturm M, Wagner AM (2010) Using repeated patterns in snow distribution modeling: An Arctic example. Water Resour Res 46: W12549. doi: 10.1029/2010WR009434.

4. Egli L, Jonas T, Gruenewald T, Schirmer M, Burlando P (2012) Dynamics of snow ablation in a small alpine catchment observed by repeated terrestrial laser scans. Hydrol Process 26: 1574-1585. doi: 10.1002/hyp.8244.
